# Environments of professional nursing practice in the context of the COVID-19 pandemic

**DOI:** 10.1097/j.pbj.0000000000000170

**Published:** 2022-02-08

**Authors:** Olga Maria Pimenta Lopes Ribeiro, Letícia de Lima Trindade, Clemente Neves de Sousa, Paulo João Figueiredo Cabral Teles, Maria Filomena Passos Teixeira Cardoso, Carla Gomes da Rocha, Sónia Cristina Costa Barros, João Miguel Almeida Ventura-Silva

**Affiliations:** a Escola Superior de Enfermagem do Porto; b Centro de Investigação em Tecnologias e Serviços de Saúde – CINTESIS, Porto, Portugal; c Universidade do Estado de Santa Catarina, Departamento de Enfermagem, Chapecó-Santa Catarina, Brasil; d Faculdade de Economia, Universidade do Porto; e LIAAD-INESC Porto LA; f Centro Hospitalar Universitário São João; g Universidade Fernando Pessoa, Porto, Portugal; h School of Health Sciences, HES-SO Valais-Wallis, Sion, Switzerland; i Instituto de Ciências Biomédicas Abel Salazar, Universidade do Porto, Porto, Portugal

**Keywords:** coronavirus infections, hospitals, nursing, pandemics, professional practice, quality assurance, health care, working environment

## Abstract

**Background:**

Although COVID-19 attracted attention to the environments of professional nursing practice, triggering a series of responses to address some of the most pressing problems, it is important to identify the size and scope of other weaknesses that have emerged.

**Methods:**

In an observational and cross-sectional study, using the Scale for the Evaluation of the Environment of Professional Nursing Practice, 752 nurses from a Portuguese university hospital, provided socio-demographic and professional data about the components structure, process and outcome of their professional nursing environment. Data collection took place from June 1–30, 2020, immediately after the first critical period of the COVID-19 pandemic in Portugal.

**Results:**

In the analysed environment of professional nursing practice, Process factors were favourable to the quality of care, while the Structure and Outcome factors had a moderately favourable effect. Structure factors related to work contexts (*P* < .001) and nursing functions in patient care areas with COVID-19 (*P* = .001). Process factors related significantly to work contexts (*P* < .001). A significant association was found between Outcome factors and work contexts (*P* < .001) and nursing functions in patient care areas with COVID-19 (*P* = .005).

**Conclusion:**

The environments of professional nursing practice in the hospital under study are moderately favourable to the quality of care. However, the need to invest in nurses’ participation, involvement and professional qualification is clear. Maintenance of a sustainable nursing workforce requires attention to be given to ensuring that practice environments are conducive to the quality of care and geared to promoting professional involvement and job satisfaction among nurses.

## Introduction

The environment of professional nursing practice, or work environment,^1^ was defined by Lake^2^ as the set of organizational characteristics that facilitate or limit professional practice. Such concept appeared in North American hospitals in the 80's and 90's, because such hospitals were known for attracting and retaining nurses and for providing high quality of care. They were called “Magnets” because they encouraged the development of favourable practice environments.^3,4^

Literature shows that a favourable environment of professional nursing practice has a positive influence on nurses such as higher levels of professional satisfaction and involvement, better perception of the quality of provided care, lower burnout levels and less intention to leave work. The impact on patients is shown by lower mortality rates and higher levels of care satisfaction. Furthermore, institutions benefit from lower nurses’ turnover and absenteeism, better quality of care and lower percentage of missed care.^4–6^

A retrospective analysis shows that environment concerns date back to the second half of the 19th century when Florence Nightingale addressed and proved the influence of the environment on patients’ recovery and showed nurses’ contributions to ensuring the quality of care.^7^

During the bicentenary of Florence Nightingale's birth, the pandemic caused by the Severe Acute Respiratory Syndrome Coronavirus 2 (SARS-CoV2), originating the infectious disease known as COVID-19, required the swift adoption by hospitals of strategies designed to provide health care and simultaneously ensure favourable environments of professional practice and professionals’ well-being.^8^

Nursing professionals around the world stand out for acting in the front lines. Therefore, nurses are considered a professional category for whom contamination, illness, death, suicide, anxiety, panic attacks and worsening of other diseases have occurred.^9,10^ In addition, nurses have been commonly responsible for organizing units to assist patients infected with the new coronavirus and, simultaneously, patients affected by other pathologies as well,^11–13^ showing great influence on the ability to support regular hospital work and proving effectiveness during the worsening of the pandemic.^12,13^ Literature has shown that nurses are aware of how to improve the environments of professional nursing practice.^14^ Currently, the COVID-19 pandemic has worsened some of the previously identified difficulties, especially in the hospital context.^7^

The fast evolution of SARS-CoV2 did not allow hospitals to appropriately prepare. Therefore, improvement strategies are required to minimize the impact of the next waves of COVID-19.

Literature also shows that quality of care can be determined by Donabedian's triad (Structure, Process and Outcome),^15^ and this triad can be used to assess the environments of professional nursing practice. To the best of our knowledge, there is no evidence of an established framework for the organization and management of the environments of professional nursing practice in the context of coping with the pandemics. However, such environments are certainly key elements in the response capacity of health services.^12^ Investment in the different dimensions of the environments of professional nursing practice will be crucial for the continuation of the fight against COVID-19. Moreover, the preparation of professionals for appropriate care practices is also important, as well as their readiness and motivation to reduce the number of COVID-19 patients.^6^

Assessment of the environments of professional nursing practice in the COVID-19 context will enable the identification of flaws and the proposal of strategies designed to improve the quality of the contexts of nursing practice.

Considering the above, the aim of this study was to analyse the Portuguese nurses’ perception of their environments of professional nursing practice in a hospital context, following the first critical period of the COVID-19 pandemic.

## Methods

An observational and cross-sectional study was carried out in an University Hospital in the North of Portugal. The used sampling technique was (non-probability) convenience sampling with a sample size of 752 registered nurses in a population of 1315. The inclusion criteria were: being a registered or specialist nurse, currently practising in the medical, surgical or intensive care departments. Professionals who were absent due to any kind of leave during the data collection period were excluded. The Strengthening the Reporting of Observational Studies in Epidemiology (STROBE) guidelines were followed.

Data were collected in June 2020, immediately after the first critical period of the COVID-19 pandemic in Portugal (between March 2 and May 15, 2020). The critical period occurred when the number of patients hospitalized for COVID-19 was the largest.

Demographic characteristics data (gender, age, marital status and academic degree) and professional characteristics data (work content, areas of care for COVID-19 patients, condition of professional nursing exercise (nurses’ role), total professional practice time, and professional practice time in the unit were collected via a questionnaire created by the authors. Information concerning the environments of professional nursing practice was collected using the Scale for the Evaluation of the Environment of Professional Nursing Practice (SEE-Nursing Practice).^16^ This scale has 93 items in three subscales: Structure (43 items with 6 dimensions); Process (37 items with 6 dimensions) and Outcome (13 items with 2 dimensions). The SEE-Nursing Practice—Structure subscale assesses the organizational factors that facilitate or hinder nurses’ work and the conditions for care providing. The SEE-Nursing Practice—Process subscale assesses the factors related to the activities concerning the design and delivery of nursing care. The SEE-Nursing Practice—Outcome subscale assesses (desirable and undesirable) changes in the quality of care and its indicators, as well as in nurses’ performance and supervision.

The Scale was arranged according to the theoretical framework by Donabedian.^15^ Scale higher scores show a more favourable environment of professional nursing practice to the quality of care. The SEE-Nursing Practice yielded a Cronbach's alpha of 0.968, 0.957, 0.916 and 0.932 respectively for the overall scale and the three subscales mentioned above.

The analysis of nurses’ perception of the environments of professional practice was described by the median, the mean and the standard deviation. In order to assess how favourable the components of the environments of professional nursing practice were to the quality of care, the following criteria were set: <35%—not very favourable; between 35% and 55%—moderately favourable; between 55% and 75%—favourable; >75%—very favourable.

In order to compare the three components and the overall scale, the Wilcoxon test was used with a significance level adjusted by the Bonferroni correction equal to 0.83% (5% nominal level). The normality of the quantitative sociodemographic and professional variables, of the overall scale and of its components was tested with the Lilliefors test. According to these results, the relationship between the sociodemographic and professional variables, the overall scale and its components was tested with the Mann-Whitney test or the Kruskal-Wallis test for qualitative variables, and Spearman's correlation coefficient for quantitative variables. The Statistical Package for the Social Sciences (version 26.0; IBM Co., USA) was used, and *P* < .05 was considered statistically significant (5% significance level) in statistical decisions.

The study was approved by the Ethics Committee of the Hospital Centre under number 137/20. Data collection was carried out by 2 researchers. In each department under study, questionnaires corresponding to the number of nurses were delivered and subsequently collected on the spot, by prior appointment and availability of the professionals. Participants signed an informed consent and were informed about the option of withdrawing without penalty. Confidentiality and anonymity in the use and disclosure of the collected data were assured.

## Results

The study involved 752 registered nurses, 76.2% were female and 63.6% were married or had a non-marital partnership. The mean age was 38 years (SD = 8.5). The majority of participants had a Bachelor's degree (87.1%), 70.2% were registered nurses and 29.8% specialist nurses. The most representative speciality area was Rehabilitation Nursing (51.8%). Participants had 15 years (SD = 8.6) of professional practice and 9.9 years (SD = 8.5) of professional practice in the current unit on average. Concerning the work context, the Department of Medicine's units were nearly half of the sample (48.5%), and half of the participants worked in areas of care for COVID-19 patients (54.5%) (Table 1).

**Table 1:** Overall sample descriptive measures and association with the work environment (n = 752)

		SEE-Nursing Practice subscales		
		
	All participants	Structure *P* value	Process *P* value	Outcome *P* value
Gender n (%)		.499^†^	.201^†^	.242^†^
Male	179 (23.8)			
Female	573 (76.2)			
Age (yr) Mean (SD)	38 (8.5)	.335^‡^	.152^‡^	.334^‡^
Marital status n (%)		.203^§^	.738^§^	.059^§^
Not married	238 (31.6)			
Married/non-marital partnership	478 (63.6)			
Divorced	32 (4.3)			
Widower	4 (0.5)			
Academic degree n (%)		.549^§^	.692^§^	.549^§^
Bachelor's degree	649 (87.1)			
Master's degree	97 (12.9)			
Work context n (%)		**<.001** ^§^	**<.001** ^§^	**<.001** ^§^
Department of Medicine Services	365 (48.5)			
Department of Surgical Services	261 (34.7)			
Department of Intensive Care Medicine	126 (16.8)			
Areas of care of COVID-19 patients	410 (54.5)	**.001** ^†^	.559^†^	**.005** ^†^
Conditions of professional nursing exercise n (%)		.125^†^	.423^†^	.778^†^
Nurse	528 (70.2)			
Specialist nurse	224 (29.8)			
Time of professional practice (yr) Mean (SD)	15 (8.6)	.232^‡^	.091^‡^	.112^‡^
Professional practice time in the service (yr) Mean (SD)	9.9 (8.5)	.141^‡^	.117^‡^	.105^‡^
Specialty area n (%)		.360^§^	.372^§^	.069^§^
Medical-Surgical Nursing	66 (29.2)			
Nursing to the Person in Palliative Situation	2 (0.9)			
Nursing to the Person in Chronic Situation	1 (0.4)			
Rehabilitation Nursing	117 (51.8)			
Mental Health and Psychiatric Nursing	3 (1.3)			
Maternal and Obstetric Health Nursing	6 (2.7)			
Child and Paediatric Health Nursing	7 (3.1)			
Community Nursing	24 (10.6)			
Nursing practice time in the specialty (yr) Mean (SD)	6 (6.3)	.115^‡^	.766^‡^	.429^‡^

SD = standard deviation.

† Mann-Whitney test

‡ Spearman.

§ Kruskal-Wallis test.

### Environments of professional nursing practice in the hospital context

Concerning the Structure subscale of the SEE-Nursing Practice, the dimensions with the highest and lowest scores were “quality and safety of nursing care” (mean = 3.4, SD = 0.8) and “nurses’ participation and involvement in the institution's policies, strategies and management” (mean = 2.8, SD = 0.9) respectively. Concerning the Process subscale, the dimensions “strategies to ensure quality in professional practice” (mean = 3.2, SD = 0.8) and “interdependent practices in professional practice” (mean = 2.5, SD = 0.8) scored below the average of the subscale (mean = 3.4, SD = 0.9). The Outcome subscale had the lowest score among all the subscales.

The Structure and Outcome subscales were moderately favourable to the quality of care, while the Process component was strongly favourable to the quality of care. Overall, the environments of professional nursing practice were moderately favourable to the quality of care (47.2%), (Table 2).

**Table 2:** Nurses’ perception of the environments of professional practice that promote quality of care

	SEE-Nursing Practice			
	
	Overall	Structure subscale	Process subscale	Outcome subscale
Dimensions, Mean (SD)		3.1 (0.9)		
People management and service leadership		3.2 (0.9)		
Physical environment and conditions for appropriate service running		3.1 (1.0)		
Nurses’ participation and involvement in the institution's policies, strategies and management		2.8 (0.9)		
Institutional policy for professional qualification		3.0 (0.8)		
Organization and guidance of nursing practice		3.2 (1.0)		
Quality and safety of nursing care		3.4 (0.8)		
Dimensions, Mean (SD)			3.4 (0.9)	
Collaboration and teamwork			3.6 (0.7)	
Strategies for ensuring quality in professional practice			3.2 (0.8)	
Autonomous practices in professional practice			3.7 (0.7)	
Care planning, evaluation and continuity			3.6 (0.8)	
Theoretical and legal support of professional practice			3.8 (0.8)	
Interdependent practices in professional practice			2.5 (0.8)	
Dimensions, Mean (SD)				2.9 (0.9)
Systematic assessment of nursing care and indicators				3.0 (0.8)
Systematic assessment of nurses’ performance and supervision				2.7 (1.0)
Environment of professional nursing practice n (%)				
Not favourable to the quality of care	26 (5.5)	84 (11.2)	7 (0.9)	140 (18.6)
Moderately favourable to the quality of care	355 (47.2)	361 (48.0)	195 (25.0)	408 (54.3)
Favourable to the quality of care	350 (46.5)	284 (37.7)	505 (67.2)	176 (23.4)
Very favourable to the quality of care	21 (2.8)	23 (3.1)	45 (6.0)	28 (3.7)

SD = standard deviation.

### Nurses’ work context and COVID-19 patient-care areas

Concerning work contexts, intensive care services have the highest scores on the overall scale and on the subscales.

Nurses who worked in COVID-19 services reported a more favourable environment of their professional practice than those who did not (Table 3). Regarding the Structure subscale, the overall average score was higher for nurses working in areas of care for COVID-19 patients (mean = 3.1, SD = 0.9) than in non-COVID-19 areas (mean = 3.0, SD = 1), with *P* = .001.

**Table 3:** Differences in the environments of professional nursing practice according to work areas

	Work areas		
		
	COVID-19	Non-COVID-19	*P* value^†^
SEE-Nursing Practice Structure, Mean (SD)	3.1 (0.9)	3.0 (1.0)	**.001**
People management and service leadership	3.2 (0.9)	3.1 (0.9)	**.043**
Physical environment and conditions for appropriate service running	3.2 (0.9)	3.0 (1.0)	**<.001**
Nurses’ participation and involvement in the institution's policies, strategies and management	2.9 (0.9)	2.8 (0.9)	**.045**
Institutional policy for professional qualification	2.5 (1.1)	2.7 (0.8)	**.015**
Organization and guidance of nursing practice	3.2 (1.0)	3.1 (1.0)	.198
Quality and safety of nursing care	3.4 (0.7)	3.4 (0.8)	.270
SEE-Nursing Practice Process, Mean (SD)	3.5 (0.8)	3.4 (0.9)	.559
Collaboration and teamwork	3.7 (0.7)	3.6 (0.8)	**.008**
Strategies for ensuring quality in professional practice	3.2 (0.8)	3.2 (0.9)	.317
Autonomous practices in professional practice	3.7 (0.7)	3.7 (0.7)	.809
Care planning, evaluation and continuity	3.5 (0.8)	3.6 (0.8)	**.031**
Theoretical and legal support of professional practice	3.8 (0.7)	3.8 (0.8)	.539
Interdependent practices in professional practice	2.5 (0.8)	2.5 (0.8)	.559
SEE-Nursing Practice Outcome, Mean (SD)	2.9 (0.9)	2.8 (0.9)	**.005**
Systematic assessment of nursing care and indicators	3.0 (0.8)	2.9 (0.8)	**.013**
Systematic assessment of nurses’ performance and supervision	2.8 (1.0)	2.7 (1.0)	**.004**

† Mann-Whitney test.

Regarding the Process subscale, nurses in COVID-19 areas reported a more favourable environment than those in non-COVID-19 areas for the ”collaboration and teamwork" dimension (mean = 3.7, SD = 0.7 and mean = 3.6, SD = 0.8 respectively; *P* = .008), unlike the “care planning, evaluation and continuity” dimension where nurses in COVID-19 areas reported less favourable environments than those in non-COVID-19 areas (mean = 3.5, SD = 0.8 and mean = 3.6, SD = 0.8 respectively; *P* = .031).

Concerning the Outcome subscale, nurses working in COVID-19 areas had higher means on the overall scale than nurses who worked in non-COVID-19 areas (mean = 2.9, SD = 0.9 and mean = 2.8; SD = 0.9 respectively; *P* = .005) (Table 3).

## Discussion

The profile of nurses who participated in the study reveals the profession's usual features with predominance of the female gender and, in the current pandemic context, a high proportion of nurses working in areas of care for COVID-19 patients.

The aspects favouring professional nursing practice in the hospital context have been studied in the last 2 decades. Among the most widely mentioned, the following stand out: appropriate human and material resources, leadership and support for nurses, actual nurses’ participation in the organization's internal policies, nursing fundamentals for the quality of care and good professional relationships.^1,4,6^

In the present study, the environments of professional nursing practice were moderately favourable to the quality of care. However, this study shows an investment in the COVID-19-assistance areas by the institution, but there are still opportunities for improvement in the COVID-19 and non-COVID-19 areas.

In the Structure Component, the dimension “nurses’ participation and involvement in the institution's policies, strategies and management” had the lowest score (mean = 2.8, SD = 0.9). A Brazilian study with 188 nurses showed the need for actions to improve working conditions, particularly concerning the opportunity that managers grant nurses to participate in the discussion of hospital issues and decisions.^3^ Another study, carried out in Brazilian hospitals during the COVID-19 pandemic and involving 104 nurses, showed that their participation in discussions about contingency planning and workflows were the most unfavourable aspects.^17^ Studies with the purpose of identifying factors that can improve nurses’ participation and involvement in the institution's policies should be carried out in the future.

Regarding the exercise of functions in patient care areas of COVID-19, the dimensions “people management and service leadership”, “physical environment and conditions for service running” and “nurses’ participation and involvement in the institution's policies, strategies and management” had the best scores in the Structure component, reflecting the fact that the investment in working conditions required by the pandemic may have contributed to a favourable perception by nurses regarding some characteristics. In fact, the concern to ensure the appropriate conditions for professional practice emerged first in patient care areas of COVID-19 in every hospital institution.^12,13^ In Portuguese hospitals, this happened in the intensive care units first.

Still in the Structure Component, the dimension “institutional policy for professional qualification” had lower scores in the patient care areas of COVID-19, highlighting the need for greater investment in a pandemic context.

The Process component stood out relatively from the others by being favourable to the quality of care. In our study, nurses’ value autonomous practices at the expense of the interdependent dimension of the profession. Therefore, despite the constraints caused by COVID-19, nurses do not neglect the design and delivery of nursing care according to the established quality standards, under the appropriate theoretical and legal support.

The comparison of nurses working in COVID-19-patient-care areas with those working in other areas showed significant differences in the dimensions “collaboration and teamwork” and “care planning, evaluation and continuity”. The average score of nurses working in COVD-19 areas was higher in the former dimension and lower in the latter. The speed of events in a pandemic context made teamwork essential,^8^ and in many situations the time to plan, to evaluate and to ensure continuity of care was scarce. In a study with 622 Spanish nurses taking care of COVID-19 patients, the need to improve communication and the relationship between the team members in order to meet the complexity of care for such patients was highlighted.^18^ Another study with 104 Brazilian nurses showed that teamwork was the most favourable aspect in the work environment during the COVID-19 pandemic in Brazil.^17^

The “systematic assessment of nurses’ performance and supervision” was the dimension with the lowest score in the Outcome subscale. The average scores in the dimensions “systematic assessment of nursing care and indicators” and “systematic assessment of nurses’ performance and supervision” were higher for nurses working in COVD-19 areas, showing their concern to assess the results obtained in contexts where professionals had to deal with COVID-19 patients on a daily basis. In the present study, intensive care nurses have a better perception of the environments of professional nursing practice than those working in medical or surgical departments. Such perception, together with the factors that may influence it, should be investigated in future studies.

The results of the application of the SEE-Nursing Practice have shown that the environments of professional nursing practice in the hospital under study are moderately favourable to the quality of care. However, the need to invest in nurses’ participation, involvement and professional qualification is clear. Furthermore, it is important to share the results of the analysis of the environments of professional nursing practice among nursing professionals in order to promote the quality and safety of care.^6,19^

Since this study was monocentric, its results cannot be generalized to other contexts. Furthermore, the study missed the responses of those professionals who were absent during the period of data collection (due to COVID-19 infection in some cases) which could have added relevant findings. Despite such limitations, it was possible to assess the environments of professional nursing practice in the pandemic context and provide detailed information on different dimensions, which are important for the quality of nursing care.

## Conclusion

The application of SEE-Nursing Practice confirmed that the environments of professional nursing practice were moderately favourable to the quality of care and showed significant associations with the work contexts and the exercise of nursing functions in areas of COVID-19 care.

This study allowed to identify which aspects of Donabedian's triad can be improved by the institution and, in this way, how to improve the quality of care and how to promote professional involvement and job satisfaction among nurses.

After returning the results, among other initiatives, it is up to the institution's managers, in nursing and other areas, to promote the participation and involvement of nurses in the institutional policies and coping strategies to deal with the COVID-19 pandemic.

## Conflicts of interest

The authors have no conflicts of interest to disclose.

## References

[1] Norman RM Sjetne IS. Measuring nurses’ perception of work environment: a scoping review of questionnaires. BMC Nurs [online serial]. 2017;16:1–15. 29200962 10.1186/s12912-017-0256-9 PMC5697362

[2] Lake ET. Development of the practice environment scale of the Nursing Work Index. Res Nurs Health [online serial]. 2002;25:176–188. 12015780 10.1002/nur.10032

[3] Yanarico DMI Balsanelli AP Gasparino RC Bohomol E. Classification and evaluation of the environment of the professional nursing practice in a teaching hospital. Rev Lat Am Enferm [online serial]. 2020;28:e3376. 10.1590/1518-8345.4339.3376 PMC7575241 33084777

[4] Wei H Sewell KA Woody G Rose MA. The state of the science of nurse work environments in the United States: a systematic review. Int J Nurs Sci [online serial]. 2018;5 3:287–300. 31406839 10.1016/j.ijnss.2018.04.010 PMC6626229

[5] Ribeiro OMPL Vicente CMFB Martins MMFPDS Vandresen L Silva JMAVD. Instruments for assessing professional nursing practice environments: An integrative review. Rev Gaucha Enferm [online serial]. 2020;41:e20190381. 32813808 10.1590/1983-1447.2020.20190381

[6] Zeleníková R Jarošová D Plevová I Janíková E. Nurses’ perceptions of professional practice environment and its relation to missed nursing care and nurse satisfaction. Int J Environ Res Public Health [online serial]. 2020;17:1–10. 10.3390/ijerph17113805 PMC7312939 32471133

[7] Ribeiro OMPL Fassarella CS Trindade LL Luna AF Silva JMAV. International year of nursing: from florence nightingale's 200th birthday to the covid-19 pandemic. Rev Enferm Cent -Oeste Min [online serial]. 2020;10:e3725.

[8] Huang L Lin G Tang L Yu L Zhou Z. Special attention to nurses’ protection during the COVID-19 epidemic. Crit Care [online serial]. 2020;24:120. 32220243 10.1186/s13054-020-2841-7 PMC7101882

[9] World Health Organization (WHO). Coronavirus Disease (COVID-19) Outbreak: Rights, Roles and Responsibilities of Health Workers, Including Key Considerations for Occupational Safety and Health [PDF]. Geneva: World Health Organization; 2021 [cited 2021 Aug 31]. Available from: https://www.who.int/docs/default-source/coronaviruse/who-rights-roles-respon-hw-covid-19.pdf?sfvrsn=bcabd401_0.

[10] Ran L Chen X Wang Y Wu W Zhang L Tan X. Risk factors of healthcare workers with coronavirus disease 2019: a retrospective cohort study in a designated hospital of Wuhan in China. Clin Infect Dis [online serial]. 2020;71:2218–2221. 32179890 10.1093/cid/ciaa287 PMC7184482

[11] David HMSL Acioli S Silva MRFD Bonetti OP Passos H. Pandemics, crisis conjunctures, and professional practices: What is the role of nursing with regard to Covid-19? Rev Gaucha Enferm [online serial]. 2020;42:e20200254. 33084792 10.1590/1983-1447.2021.20190254

[12] Huang LH Chen CM Chen SF Wang HH. Roles of nurses and National Nurses Associations in combating COVID-19: Taiwan experience. Int Nurs Rev [online serial]. 2020;67:318–322. 32761608 10.1111/inr.12609 PMC7436573

[13] Zhang Y. Strengthening the power of nurses in combating COVID-19. J Nurs Manag. 2021;29:357–359. 32259325 10.1111/jonm.13023 PMC7262074

[14] Ribeiro OMPL Vicente CMFB Martins MMFPS Trindade LL Sousa CN Cardoso MFPT. Scale of evaluation of the environments of professional nursing practice: construction and content validation. Rev Baiana enferm [online serial]. 2020;34:e37996.

[15] Donabedian A. An Introduction to Quality Assurance in Health Care. New York: Oxford University Press; 2003.

[16] Ribeiro OMPL Vicente CMFDB Sousa CN Teles PJFC Trindade LL Martins MMFPDF Scale for the environment evaluation of professional nursing practice: construct validation. J Nurs Manag [online serial]. 2021;1–10. 33605488 10.1111/jonm.13290

[17] Santos JLGD Balsanelli AP Freitas EO Menegon FHA Carneiro IA Lazzari DD Work environment of hospital nurses during the COVID-19 pandemic in Brazil. Int Nurs Rev [online serial]. 2021;68:228–237. 33586794 10.1111/inr.12662 PMC8014554

[18] González-Gil MT González-Blázquez C Parro-Moreno AI Nurses’ perceptions and demands regarding COVID-19 care delivery in critical care units and hospital emergency services. Intensive Crit Care Nurs [online serial]. 2021;62:102966. 33172732 10.1016/j.iccn.2020.102966 PMC7598734

[19] Falguera CC Santos JA Galabay JR Relationship between nurse practice environment and work outcomes: a survey study in the Philippines. Int J Nurs Pract [online serial]. 2020;27:e12873. 32677223 10.1111/ijn.12873

